# Vital Signs Directed Therapy for the Critically Ill: Improved Adherence to the Treatment Protocol Two Years after Implementation in an Intensive Care Unit in Tanzania

**DOI:** 10.1155/2020/4819805

**Published:** 2020-01-07

**Authors:** Anna Hvarfner, Jonas Blixt, Carl Otto Schell, Markus Castegren, Edwin R. Lugazia, Moses Mulungu, Helena Litorp, Tim Baker

**Affiliations:** ^1^Medical Faculty, Uppsala University, Uppsala, Sweden; ^2^Mora Hospital, Region Dalarna, Mora, Sweden; ^3^Center for Clinical Research Dalarna, Uppsala University, Falun, Sweden; ^4^Perioperative Medicine and Intensive Care, Karolinska University Hospital, Stockholm, Sweden; ^5^Department of Physiology and Pharmacology, Karolinska Institute, Stockholm, Sweden; ^6^Department of Global Public Health, Karolinska Institutet, Stockholm, Sweden; ^7^Department of Internal Medicine, Nyköping Hospital, Region Sörmland, Nyköping, Sweden; ^8^Centre for Clinical Research Sörmland, Uppsala University, Eskilstuna, Sweden; ^9^CLINTEC, Karolinska Institute, Stockholm, Sweden; ^10^Department of Anaesthesiology, Muhimbili National Hospital, Dar es Salaam, Tanzania; ^11^Department of Anaesthesiology, Muhimbili University of Health and Allied Science, Dar es Salaam, Tanzania; ^12^International Maternal and Child Health, Department of Women's and Children's Health, Uppsala University, Uppsala, Sweden; ^13^College of Medicine, Blantyre, Malawi

## Abstract

Treating deranged vital signs is a mainstay of critical care throughout the world. In an ICU in a university hospital in Tanzania, the implementation of the Vital Signs Directed Therapy Protocol in 2014 led to an increase in acute treatments for deranged vital signs. The mortality rate for hypotensive patients decreased from 92% to 69%. In this study, the aim was to investigate the sustainability of the implementation two years later. An observational, patient-record-based study was conducted in the ICU in August 2016. Data on deranged vital signs and acute treatments were extracted from the patients' charts. Adherence to the protocol, defined as an acute treatment in the same or subsequent hour following a deranged vital sign, was calculated and compared with before and immediately after implementation. Two-hundred and eighty-nine deranged vital signs were included. Adherence was 29.8% two years after implementation, compared with 16.6% (*p* < 0.001) immediately after implementation and 2.9% (*p* < 0.001) before implementation. Consequently, the implementation of the Vital Signs Directed Therapy Protocol appears to have led to a sustainable increase in the treatment of deranged vital signs. The protocol may have potential to improve patient safety in other settings where critically ill patients are managed.

## 1. Introduction

With several million deaths per year, there is a large global burden of critical illness [1]. Low- and middle-income countries have a disproportionally high share with over 90% of global trauma deaths and deaths from pneumonia, meningitis, and other infections [1–3]. Yet, there is a lack of knowledge about how critical care is optimally performed in low-resource settings [4]. Worldwide, intensive care units (ICUs) are used for the care of critically ill patients, with a high nurse: patient ratio and a focus on monitoring vital signs and supporting failing organ functions [5]. Many low-income countries (LICs) lack data on ICU capacity, and countries with available data have few ICU beds per capita [6, 7]. There is a shortage of ICU physicians, and most care is performed by nurses and health care personnel who lack formal specialised training [8]. Patients admitted to ICUs in LICs have a higher risk of in-hospital death than patients in high-income countries [9].

**Figure 1:**
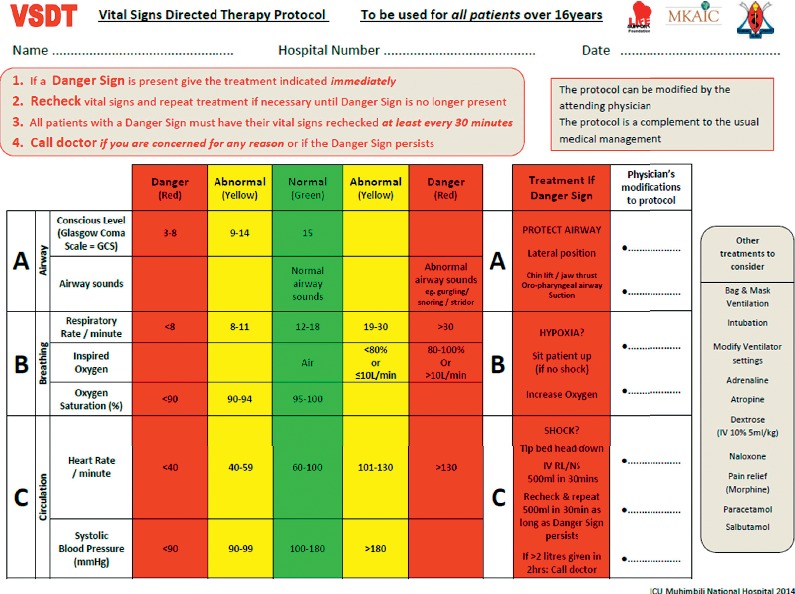
The Vital Signs Directed Therapy Protocol. The protocol provides criteria for the classification of vital signs as either normal (green), abnormal (yellow), or danger sign (red) and prompts treatments for danger signs. It was introduced in the main intensive care unit in Muhimbili National Hospital in March 2014.

In the ICU in Muhimbili National Hospital (MNH), Tanzania, single deranged vital signs were found to be associated with mortality but rarely followed by an acute intervention [10, 11]. To improve care, the Vital Signs Directed Therapy (VSDT) Protocol (Figure 1) was introduced [12]. In the VSDT protocol, vital signs are classified as either normal (green), abnormal (yellow), or danger sign (red). A danger sign prompts an immediate treatment, as suggested in the protocol. To enable quick care in the absence of a physician, the VSDT protocol was designed to include actions that could be task-shifted and carried out independently by nurses. Task-shifting has been used for decades as a method of expanding access to care [13–16] and has been successful in emergency care in Uganda [17] and for identifying critically ill paediatric patients in Malawi [18].

The VSDT protocol was implemented in the main ICU in MNH in March 2014 using a multimodal approach which involved all nurses and doctors working in the unit [12]. Two doctors and four nurses were local facilitators, designated to reinforce the use of the protocol after the implementation period, and received extra training. A similar approach had previously been found successful in promoting adherence to clinical guidelines in paediatric admissions in Kenya [19].

The VSDT protocol improved acute treatments of danger signs: the proportion of all danger signs that were followed by an acute treatment in the ICU increased from 2.9% before implementation to 16.6% immediately after implementation (*p* < 0.001) [12]. Mortality was reduced for patients with hypotension (from 92.3% to 69.2%, *p*=0.02) but was unchanged for all other patients. Following the implementation period, the use of the VSDT protocol continued, but there were no further inputs from the research team.

The aim of this study was to investigate the sustainability of the VSDT implementation in MNH through determining adherence to the VSDT protocol two years after implementation.

## 2. Methods

We conducted an observational, patient-record-based study of deranged vital signs and acute treatments in the main ICU in MNH between August 4^th^ and August 31^st^, 2016. The study was a follow-up study to the original implementation study—the results from this study were compared with the results from the implementation study [12].

### 2.1. Setting

Tanzania is a LIC in East Africa with a population of 55.6 million and a physician density of 0.02 per 1000 people [20, 21]. While drugs and equipment for basic critical care are generally available in Tanzanian hospitals, a deficit in infrastructure and routines for good quality critical care have been shown [22].

MNH is a national referral and university teaching hospital with 1,500 beds, receiving patients from all over Tanzania [23]. The study took place in the six-bedded main ICU where the VSDT protocol had already been introduced through a multimodal approach including sensitisation, training, job aids, supervision, and feedback in 2014 [12]. During the time of the study, nurses worked in 12-hour shifts, seven nurses in the day and four nurses at night. Vital signs were assessed hourly for every patient according to established routines. Oxygen, fluids, and antibiotics were in stock, and each bed had a mechanical ventilator. There were no temperature cables, cardiac monitors, fentanyl, morphine, or midazolam.

### 2.2. Data Collection

The patients' handwritten ICU observations charts and the admission book were photographed every day by the same group of ICU nurses as in the implementation study [12]. Patients were followed until hospital discharge or death and outcome data were recorded.

All documented vital signs observations from patients >16 years of age admitted to the ICU between August 4^th^ and August 31^st^, 2016 were screened. Danger signs and acute treatments according to the VSDT protocol (Figure 1) were extracted from the charts twice (double data entry) and any discrepancies between the two data sets resolved by returning to the source data. The time-of-day when a danger sign was detected was noted to compare practices in the mornings (07:00–14:59), afternoons (15:00–22:59), and nights (23:00–06:59). For each chart, the total number of observations were recorded. Information on patient characteristics was extracted from the admission book and follow-up records, and diagnoses were grouped into categories.

A telephone interview with one of the ICU nurses was performed to collect information about changes to the ICU and the hospital in general since the protocol was implemented. The same questionnaire was used as in the implementation study [12].

### 2.3. Outcome Measure and Definition of Variables

The outcome measure was adherence to the VSDT protocol, defined as the stipulated treatment in the VSDT protocol carried out in the same hour or subsequent hour following a danger sign. Danger signs were defined by the criteria in the VSDT protocol. As in the implementation study, some danger signs and treatments were excluded from the analysis as they either had no corresponding directive in the VSDT protocol or there was a lack of sufficient documentation. The excluded danger signs and treatments were as follows: a low conscious level in patients with an endotracheal tube in situ; a high level of inspired oxygen; deranged respiratory rate or level of oxygen saturation in patients already receiving 100% oxygen; position of the patient; chin lift/jaw thrust; oropharyngeal airway; and suction [12].  Table 1 shows danger signs and acute treatments that were included in the analysis.

### 2.4. Statistical Analysis

Median and range were used for descriptive analyses of non-normally distributed variables. Adherence in this study was compared with the adherence in the original implementation study using chi-squared tests for categorical data or Fischer's exact tests when the expected cell count was less than five. *p* < 0.05 was considered statistically significant. Confidence intervals of 95% were calculated. The analyses were done using SPSS (SPSS Statistics Version 25 IBM).

### 2.5. Ethical Considerations

Ethical clearance was granted by the National Institute for Medical Research in Tanzania (NIMR/HQ/R.8a/Vol.IX/1606), Muhimbili University of Health & Allied Sciences (MU/DRP/AEC/Vol.XVI/125), and the Ethical Review Board in Stockholm, Sweden (EPN/2015/673-31/2). As the study was part of quality improvement in the ICU and data were anonymized before analysis, individual patient consent was waived.

## 3. Results

### 3.1. Danger Signs

A total of 1922 vital sign observations were assessed and 289 danger signs were included in the analysis (Table 2). Danger signs for bradycardia and bradypnea were found on only one occasion each. All other danger signs for heart rate were tachycardia, and for respiratory rate were tachypnea.

### 3.2. Patient Characteristics

During the study period, 21 adult patients were cared for in the ICU. For three patients, no observation charts were found. For the rest of the patients (*n* = 18), all the charts were used except for five charts that were missing for two patients. Consequently, the danger signs included in this study came from 18 ICU patients and 94 observation charts (Table 3). On admission, ten patients (55.6%) had at least one danger sign (range 0–3), 14 (77.8%) were receiving oxygen therapy, and 14 (77.8%) had an endotracheal tube in situ.

### 3.3. Adherence to the VSDT Protocol

**Figure 2:**
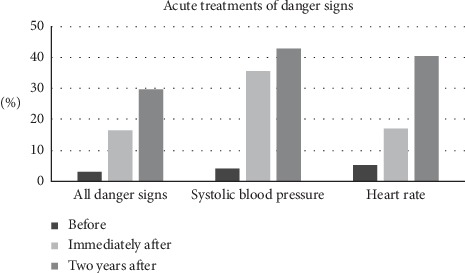
Acute treatments of danger signs. Proportion of danger signs that were followed by an acute treatment according to the Vital Signs Directed Therapy Protocol before implementation, immediately after implementation [12], and two years after implementation (shown for all danger signs combined and for the systolic blood pressure danger sign and heart rate danger sign separately).

Adherence for all danger signs two years after implementation was 29.8% (95% CI 24.7–35.2%). This was higher than the adherence immediately after implementation (16.6% (95% CI 14.7–18.7%), *p* ≤ 0.001) and higher than the proportion of danger signs that received an acute treatment as stipulated in the protocol before implementation (2.9%, *p* ≤ 0.001) (Table 4, Figure 2) [12]. Adherence for each danger sign varied, from no adherence for the oxygen saturation danger sign to 100% adherence for the conscious level danger sign. Compared with immediately after implementation, there was a significant increase in adherence for the heart rate danger sign (from 17.1% to 40.4%, *p* ≤ 0.001); there were no significant differences in adherence for the systolic blood pressure, the oxygen saturation, and the respiratory rate danger signs. No comparison of adherence to the immediately-after-implementation group was done for the conscious level danger sign, as there was no danger sign present in this group. The increase in adherence for all danger signs remained even if the heart rate danger sign was removed from the analysis. Compared with before implementation, there were significant increases in acute treatments of the systolic blood pressure danger sign (from 4.1% to 42.9%, *p* ≤ 0.001) and conscious level danger sign (from 3.8% to 100%, *p* ≤ 0.001).

Adherence two years after implementation was significantly higher in the mornings (40.2%) compared with that in the afternoons (25.7%, *p* < 0.05) and nights (25.5%, *p* < 0.05). There was no significant difference in adherence between afternoons and nights.

## 4. Discussion

Adherence to the VSDT protocol was 29.8% two years after implementation in an ICU in a national referral hospital in Tanzania. This was significantly higher than in the period immediately after implementation (16.6%) and before implementation (2.9%).

The increased adherence to the protocol two years after implementation may be a surprise, as other studies have shown decreasing effects of clinical interventions over time [24, 25]. In Rwanda, adherence to successfully implemented handwashing guidelines decreased from 69% to 37% in the year following implementation [26]. Our results could indicate that the multimodal approach chosen for the implementation of the VSDT protocol was successful in making sustainable changes [12]. Appointing local facilitators was also associated with a high sustainment of an antiretroviral therapy scaleup program in Uganda [27]. Other factors may have affected the results, such as changes to the patient population or routines in the ICU. Most patients in this study were admitted to the ICU following elective surgery, while the patient groups before and immediately after implementation were more diverse in terms of diagnoses [12]. Furthermore, there was an increased number of ICU beds in the hospital following the opening of a medical ICU in March 2016 and an ICU for women with eclampsia in August 2016.

The higher adherence in the mornings compared with afternoons and nights two years after implementation could be due to the influence of doctors' ward rounds in the mornings or that it is easier to contact a doctor in the morning than during the rest of the day. The same results were also found in the period immediately after implementation [12].

Despite the increased adherence, 70.2% of the observed danger sings in this study were not followed by the recommended treatment. This could signify that some staff do not understand the severity of danger signs or that some staff do not know how to use the VSDT protocol or alternatively that treatments were not possible because resources were lacking. The VSDT protocol was developed as a compliment to the usual medical management to enable quick emergency care when access to a physician is limited [12]. Once a physician is present, his/her medical judgement may supersede the protocol and other, more advanced, treatments might be chosen instead, such as modification of ventilator settings or administration of medications. Staff can also have good reasons to refuse treatments prompted by the protocol, for example to avoid harmful fluid overload [28]. An adherence to the protocol that was close to 100% may even indicate that some patients would receive inappropriate treatments.

Due to the limited number of patients in this study, comparative analyses of patient outcomes were not appropriate and so the effect of the increased treatment of danger signs remains unclear. However, actions suggested in the VSDT protocol are usually part of routine care for deranged physiology and have been shown to prevent deterioration and death in other settings [29]. The original implementation of the VSDT protocol in the MNH ICU resulted in a reduction of in-hospital deaths, from 92% to 69%, for hypotensive patients. Overall in-hospital mortality for ICU patients in the original study was 49%, and among the 18 patients in this study, it was 39%. Potential beneficial effects of the VSDT protocol on patient outcome need to be evaluated in a larger study but could possibly include reductions in mortality and ICU-length of stay as deteriorating patients are identified and given simple but crucial treatments early.

The strengths of this follow-up study were the use of the same methodology as in the implementation study and the VSDT protocol itself which was designed to be context-appropriate, pragmatic, and sustainable with a focus on the essential management of critical illness [30]. This study also has several limitations. Due to logistical restraints, the study period of one month was short and included data from only 18 patients, making the results vulnerable to temporal trends. The staff were aware that the charts were being photographed, which potentially encouraged improved adherence to the protocol—a Hawthorn effect. We do not believe that this affected our results, as the staff were used to the photographing process from the previous two-year implementation study. The photographs of the handwritten charts were of variable quality and sometimes hard to interpret. We minimized the effect of potential misreadings by performing double data entry. Generalizing conclusions from this study should be done with caution.

Future studies should evaluate the effect on patient outcomes of an improved adherence to the protocol and assess the cost-effectiveness of the VSDT-implementation. Qualitative research on factors affecting the use of the VSDT protocol are also of importance to further optimize its design and methods for implementation.

## 5. Conclusion

Adherence to the Vital Signs Directed Therapy (VSDT) Protocol in a Tanzanian ICU increased two years after implementation. The protocol, following a multimodal implementation, may be able to contribute to a sustainable improvement in the treatment of deranged vital signs in critical illness. As the VSDT actions are pragmatic and cheap, the protocol may have potential to improve patient safety in other low-resourced or low-staffed settings where critically ill patients are managed.

## Figures and Tables

**Table 1:** Danger signs and associated actions that were included in the analysis of adherence.

Danger sign	Action(s)
Conscious level (Glasgow coma scale 3–8)	Endotracheal intubation
Respiratory rate (<8 or >30 breaths per minute)	Initiation/increase of oxygen therapy
Oxygen saturation (<90%)	
Heart rate (<40 or >130 beats per minute)	Administration of 500 ml intravenous ringer lactate or normal saline
Systolic blood pressure (<90 mmHg)	

**Table 2:** Distribution of danger signs.

Distribution of danger signs	*n* (%)
Any danger sign	289
Systolic blood pressure (<90 mmHg)	84 (29.1)
Heart rate (<40 or >130 beats per minute)	114 (39.4)
Oxygen saturation (<90%)	1 (0.3)
Conscious level (Glasgow coma scale 3–8)	3 (1.0)
Respiratory rate (<8 or >30 breaths per minute)	87 (30.1)

**Table 3:** Patient characteristics.

	*n* (%) *N* = 18
Females	9 (50.0)
Age, median (range)	50.0 (21–75)
Days in ICU, median (range)	3 (0–23)
Died in-hospital	7 (38.9)
Diagnosis type	
Elective surgery	10 (55.6)
Acute surgery	1 (5.6)
Trauma/burn/foreign body	3 (16.7)
Obstetrics	1 (5.6)
Internal medicine/infection	2 (11.1)
Unknown	1 (5.6)

ICU: intensive care unit.

**Table 4:** Adherence to the Vital Signs Directed Therapy Protocol immediately after implementation [12] and two years after implementation.

	Immediately after implementation (12)	Two years after implementation	*p* value
All danger signs	16.6% (*n* = 1299) (95% CI 14.7–18.7%)	29.8% (*n* = 289) (95% CI 24.7–35.2%)	<0.001^*∗*^
Systolic blood pressure	35.0% (*n* = 306)	42.9% (*n* = 84)	0.184^*∗*^
Heart rate	17.1% (*n* = 549)	40.4% (*n* = 114)	<0.001^*∗*^
Oxygen saturation	16.0% (*n* = 25)	0% (*n* = 1)	—
Conscious level	(*n* = 0)	100% (*n* = 3)	—
Respiratory rate	2.6% (*n* = 419)	1.1% (*n* = 87)	0.701^*∗∗*^
All, except heart rate, danger signs	16.3% (*n* = 750)	22.9% (*n* = 175)	0.039^*∗*^

^*∗*^Calculated with the chi-squared test, ^*∗∗*^calculated with Fisher's exact test. CI confidence interval.

## Data Availability

Due to ethical and legal reasons, e.g., since the numbers of individuals in this study was small, data cannot be shared publicly as it would compromise patient confidentiality. Anonymized data can be provided after contact with the researcher.

## Abbreviations

VSDT: Vital Signs Directed Therapy
LIC: Low-income country
MNH: Muhimbili National Hospital.
